# Engineering a dual vaccine against COVID-19 and tuberculosis

**DOI:** 10.3389/fcimb.2023.1273019

**Published:** 2023-10-26

**Authors:** Carlyn Monèt Guthrie, Xuejuan Tan, Amber Cherry Meeker, Ashton Elisabeth Self, Lin Liu, Yong Cheng

**Affiliations:** ^1^ Department of Biochemistry and Molecular Biology, Oklahoma State University, Stillwater, OK, United States; ^2^ Oklahoma Center for Respiratory and Infectious Diseases, Oklahoma State University, Stillwater, OK, United States; ^3^ Department of Physiological Sciences, Oklahoma State University, Stillwater, OK, United States

**Keywords:** COVID-19, tuberculosis, *Mycobacterium bovis* BCG, dual vaccine, mice

## Abstract

The COVID-19 pandemic, caused by SARS-CoV-2 virus, has been one of the top public health threats across the world over the past three years. *Mycobacterium bovis* BCG is currently the only licensed vaccine for tuberculosis, one of the deadliest infectious diseases in the world, that is caused by *Mycobacterium tuberculosis*. In the past decades, recombinant *M.bovis* BCG has been studied as a novel vaccine vector for other infectious diseases in humans besides tuberculosis, such as viral infections. In the current study, we generated a recombinant *M. bovis* BCG strain AspikeRBD that expresses a fusion protein consisting of *M. tb* Ag85A protein and the receptor-binding domain (RBD) of the SARS-CoV-2 spike protein using synthetic biology technique. Our results show that the recombinant *M. bovis* BCG strain successfully expressed this fusion protein. Interestingly, the recombinant *M. bovis* BCG strain AspikeRBD significantly induced SARS-CoV-2 spike-specific T cell activation and IgG production in mice when compared to the parental *M.bovis* BCG strain, and was more potent than the recombinant *M.bovis* BCG strain expressing SARS-CoV-2 spike RBD alone. As expected, the recombinant *M. bovis* BCG strain AspikeRBD activated an increased number of *M. tb* Ag85A-specific IFNγ-releasing T cells and enhanced IgG production in mice when compared to the parental *M.bovis* BCG strain or the BCG strain expressing SARS-CoV-2 spike RBD alone. Taken together, our results indicate a potential application of the recombinant *M. bovis* BCG strain AspikeRBD as a novel dual vaccine against SARS-CoV-2 and *M. tb* in humans.

## Introduction

Bacteria-vectored vaccines are a type of vaccines that use attenuated live bacteria as the carrier to safely deliver antigens into the host, which will then induce antigen-specific immune responses in the host and provide robust protection against future infection and/or diseases (Chen et al., 2012; Lin et al., 2015; Chauchet et al., 2016). *Mycobacterium bovis* bacillus Calmette-Guérin (BCG), an attenuated *Mycobacterium* strain, was first administered to humans to prevent tuberculosis (TB) caused by bacterial pathogen *M. tuberculosis* (*M. tb*). After over a century, BCG still remains the only available vaccine against TB and has been administered to more individuals than any other vaccine in the world with an undoubted safety record (Philips and Ernst, 2012; Andersen and Scriba, 2019). Compared to other common vaccines, the BCG vaccine demonstrates well-proven safety, excellent stability, convenience for mass production and transportation, affordability and accessibility for low- and middle-income countries and, very importantly, a reliable immunogenicity profile (Gonzalez-Perez et al., 2021; Mouhoub et al., 2021). Inspired by these outstanding features, BCG has also been widely studied as a bacterial vector for vaccine delivery system against infectious diseases caused by non-mycobacterial pathogens such as human immunodeficiency virus (HIV) (Chapman et al., 2010; Kim et al., 2018; Kilpeläinen et al., 2019), hepatitis C virus (Uno-Furuta et al., 2003), human metapneumovirus (Palavecino et al., 2014), human respiratory syncytial virus (Rey-Jurado et al., 2017), rotavirus VP6 (Dennehy et al., 2007), *Bordetella pertussis* (Nascimento et al., 2000), *Borrelia burgdorferi* (the causative agent of Lyme disease) (Stover et al., 1993), *Plasmodium* (the causative parasite of malaria) (Matsumoto et al., 1998) and measles virus (Zhu et al., 1997). These studies confirm that recombinant BCG strains can stimulate a robust microbe-specific immune response in the host, thereby protecting the host from the corresponding microbial infection. Thus, it is notable that recombinant BCG strains may represent a desirable vaccine platform for the development of safe and effective anti-microbial vaccines.

Coronavirus disease 2019 (COVID-19) is caused by severe acute respiratory syndrome coronavirus 2 (SARS-CoV-2) and has become one of the top threats to both public health and global economics. Vaccination has undoubtedly saved millions of lives and been proven to be a critical tool to mitigate this pandemic. Currently, lipid nanoparticles or modified adenovirus are popular delivery vehicles for licensed COVID-19 vaccines (Heinz and Stiasny, 2021). To better control the COVID-19 pandemic, there is still an urgent need to develop a novel COVID-19 vaccine platform with potent efficacy and improved safety characteristics. Thus, a well-investigated and long-term clinically proven vaccine delivery technique, the bacteria-based vector, appears to be an attractive option.

COVID-19 and TB are currently two of the most fatal infectious diseases in the world. Over the past three years, the COVID-19 pandemic has resulted in an estimated 15 million deaths across the world (Barouch, 2022; Edwards et al., 2022). On the side of TB, according to the WHO annual report, it is estimated that one quarter of the global population, which corresponds to about two billion people, has been infected by *M. tb*. Without treatment, about 5-10% of infected individuals will progress to active TB during their lifetime, leading to approximately 10 million active TB cases and 1.5 million deaths annually. In 2020, the COVID-19 pandemic unprecedentedly increased TB deaths and reversed years of global progress in reducing TB mortality (WHO, 2021). Both COVID-19 and TB are respiratory infectious diseases that damage primarily the lungs but also attack many other organs. Considering their similar symptoms, clinical parameters, and especially immunopathogenic characteristics (Sheerin et al., 2022; The TB/COVID-19 Global Study Group, 2022), it is highly feasible to develop a novel dual-vaccine targeting these two related infectious diseases simultaneously (Jia et al., 2021). Additionally, the robust immunogenicity profile of a recombinant BCG strain carrying viral protein antigens (such as HIV) indicates the potential of BCG as a potent platform for the development of novel COVID-19 vaccines (Chapman et al., 2010; Kim et al., 2018; Kilpeläinen et al., 2019). Therefore, we hypothesize that BCG can be safely used as a vector to deliver both SARS-CoV-2 and *M. tb* specific epitopes into human cells thereby generating a reliable and potent dual vaccine against COVID-19 and TB simultaneously.

In our previous study, we developed a novel BCG-based vaccine platform and verified that this delivery system could efficiently activate exosome-dependent antigen presentation in innate immune cells and subsequently induce antigen-specific immune responses in the lungs of mice after vaccination (Smith et al., 2017). Based on the similar principle, in our current study, we generated a dual vaccine candidate, *M. bovis* BCG AspikeRBD, against COVID-19 and TB simultaneously using BCG as a vaccine vector. *M. bovis* BCG AspikeRBD is a recombinant BCG strain that constitutively expresses a fusion protein consisting of the *M. tb* Ag85A protein and the SARS-CoV-2 spike receptor-binding domain (RBD). The Ag85A protein is a member of the antigen 85 complex that plays an important role in the integrity of cell wall in *M. tb* (Ronning et al., 2004) . SARS-CoV-2 spike protein is essential for viral binding to and infecting the airway epithelium via the interaction between its RBD and human cell surface protein angiotensin-converting enzyme 2 (ACE2) (Wrapp et al., 2020; Yang and Rao, 2021). Our results demonstrated that *M. bovis* BCG AspikeRBD significantly induced *M. tb* Ag85A- and SARS-CoV-2 spike-specific IgG production in the serum and T-cell activation in the lungs and spleen of immunized mice. Compared with a recombinant BCG strain expressing the SARS-CoV-2 spike RBD alone, *M. bovis* BCG AspikeRBD induced much stronger spike-specific immune responses. Taken together, our data suggest that the recombinant BCG strain, *M. bovis* BCG AspikeRBD, may potentially work as a novel dual vaccine for both SARS-CoV-2 and *M. tb* infection.

## Materials and methods

### Animals

Wild-type C57BL/6 mice were purchased from Jackson Laboratory (Stock # 000664) and housed at the institutional animal facility under specific pathogen-free conditions at Oklahoma State University, which are accredited through the Animal Welfare Assurance (# A3722-01). All animal experiments were approved by the Institutional Animal Care and Use Committees (IACUC) of Oklahoma State University.

### Bacterial strains

*M. bovis* BCG strains were grown in Middlebrook 7H9 broth medium (Cat. No. M198, HiMedia Laboratories) containing 10% OADC (oleic acid/albumin/dextrose/catalase, 0.05% Tween 80) as we did previously (Cheng and Schorey, 2013; Smith et al., 2017; Cheng and Schorey, 2018) until exponential phase and then aliquoted and stored at − 80°C until use. Prior to use, the bacterial stocks were thawed and the mycobacteria were de-clumped by a brief sonication and passed through a syringe fitted with a 27-gauge needle.

### Generation of plasmids

The plasmid pMV261-Ag85A::SpikeRBD and pMV261-SpikeRBD were generated using a fast-cloning technique as described previously (Li et al., 2011). The pMV261 vector was amplified using the primer pMV261-FW and pMV261-RE (**Table 1**) from pMV261-DsRed (Smith et al., 2017) with Phusion^®^ High-Fidelity DNA Polymerase (Cat. No. M0530L, New England Biolabs). Similarly, the DNA fragments encoding *M.tb* Ag85A and SARS-CoV-2 spike RBD were amplified by PCR from the plasmid pMV261-Ag85A::DsRed (Smith et al., 2017) and the vector pHDM (Cat. No. NR-52514, BEI Resources), respectively. The pMV261-Ag85A::SpikeRBD plasmid was generated using the pMV261 vector (Primers: pMV261-FW and pMV261-RE) and the DNA fragments of Ag85A (Primers: Ag85A-FW and Ag85A-RE) and SpikeRBD (Primers: SC2-SpikeRBD-FW1 and SC2-SpikeRBD-RE) (**Table 1**). The pMV261-SpikeRBD plasmid was generated using the pMV261vector (Primers: pMV261-FW and pMV261-RE) and the DNA fragment of SpikeRBD (Primers: SC2-SpikeRBD-FW2 and SC2-SpikeRBD-RE) (**Table 1**). The overlapping sequences of each PCR fragment are underlined in the primers as shown in **Table 1**. The PCR products were mixed at a ratio of 1:1:1 (PMV261:Ag85A:SpikeRBD) or 1:1 (PMV261:SpikeRBD) and incubated at room temperate for 5 min before transformed into *E.coli* DH5α. The resulting plasmids were purified and verified by PCR and further confirmed by DNA-sequencing analysis in the Genomics Core Facility at Oklahoma State University.

**Table 1:** Primers used in this study.

Primers	Sequence (5’ -> 3’)
pMV261-FW	**TAGGTTAACTAGCGT** ACGATCGACTGC
pMV261-RE	**CAT** GGATCCGCAATTGTCTTGGCCA
Ag85A-FW	GACAATTGCGGATCC **ATG** CAGCTTG
Ag85A-RE	*GCTAGCAGGTCCGGC* GCCC
SC2-SpikeRBD-FW1	*GCCGGACCTGCTAGC* GAGAAAGGCATTTATCAGACTTCTAACT
SC2-SpikeRBD-FW2	AATTGCGGATCC **ATG** GAGAAAGGCATTTATCAGACTTCTAACT
SC2-SpikeRBD-RE	**ACGCTAGTTAACCTA** CACGCCTGTTCCAGTCAGC

### Electroporation of mycobacteria

The plasmids pMV261-Ag85A::SpikeRBD and pMV261- SpikeRBD were respectively transformed into wild-type *M. bovis* BCG as we did previously (Smith et al., 2017; Cheng and Schorey, 2018). The transformed *M. bovis* BCG colonies were selected on Middlebrook 7H10 agar plates (Cat. No. M199, HiMedia Laboratories) supplemented with 0.5% glycerol, 10% OADC and 50 μg/mL of hygromycin at 37°C. Hygromycin-resistant colonies were sub-cultured and confirmed by PCR using pMV261-specific primers and Western blot for SARS-CoV-2 spike protein. Resulting *M. bovis* BCG strains were named as *M. bovis* BCG AspikeRBD and *M. bovis* BCG SpikeRBD, respectively.

### Western blot

The assay was performed as we did previously (Cheng and Schorey, 2016; Cheng and Schorey, 2019; Cheng et al., 2020). The membrane was incubated with rabbit antibody against SARS-CoV-2 spike protein at 1:1000 dilution (Cat. No. NR-52947, BEI Resources), followed by the secondary goat anti-rabbit HRP-conjugated IgG (Cat. No. 31460, Invitrogen).

### Immunization in mice

Wild-type C57BL/6 mice (6 weeks old, Female) were subcutaneously immunized with a single dose (1.0 x 10^6^ CFU/mouse) of parental *M. bovis* BCG, *M. bovis* BCG AspikeRBD or *M. bovis* BCG SpikeRBD strain in 100 μL of PBS.

### Lymphocyte isolation and ELISPOT

Mouse lung and spleen cells were isolated 4 weeks after *M. bovis* BCG immunization as we did previously (Cheng and Schorey, 2013). The ELISPOT assay for IFNγ-releasing T-cells was performed as we did previously (Cheng and Schorey, 2016) using anti-mouse IFNγ capture antibody (Cat. No. 505701, Biolegend) and biotin-conjugated detection antibody (Cat. No. 505803, Biolegend), followed by avidin-conjugated HRP (Cat. No. 405103, Biolegend). Mouse lung and spleen cells (2.0 x 10^5^ cells/well) were analyzed 24 h after *ex vivo* individually re-stimulated with recombinant SARS-CoV-2 full spike protein (Cat. No. NR-52397, Bei Resources), spike RBD (Cat. No. NR-52307, BEI Resources), or *M. tb* Ag85A (Cat. No. NR-53525, BEI Resources).

### Antibody endpoint titers

Mouse blood was collected 4 weeks after *M. bovis* BCG immunization and antigen-specific IgG titers were determined as we described previously (Cheng and Schorey, 2013). Briefly, Nunc Polysorp plates were coated with recombinant SARS-CoV-2 spike RBD (Cat. No. NR-52307, BEI Resources) or *M. tb* Ag85A (Cat. No. NR-53525, BEI Resources) at 2 μg/mL in 0.1 M bicarbonate solution overnight at 4°C. Antigen-coated plates were then blocked with 0.05% PBS-tween 20 plus 1% BSA for 2 h at room temperature. Mouse sera were added into the plates and incubated for another 2 h at room temperature. Plates were washed and treated with goat anti-mouse IgG HRP (Cat. No. G21040, Invitrogen) for 1 h at room temperature. Plates were washed and developed with tetramethylbenzidine (Cat. No. 00-4201-56, Invitrogen) and the reaction was stopped by adding 1 N H_2_SO_4_. Finally, plates were read and analyzed using BioTek Synergy H1 plate reader at 450 nm and BioTek Gen5 software with a cutoff of 0.1 absorbance value since negative controls have OD values close to 0.1.

### Statistical analysis

The data obtained were analyzed by Student paired t-test. A value of p ≤ 0.05 was considered significant. The computer program GraphPad PRISM 9.5.0 was used for the analysis.

## Results

### Generation and evaluation of recombinant BCG strains

**Figure 1:**
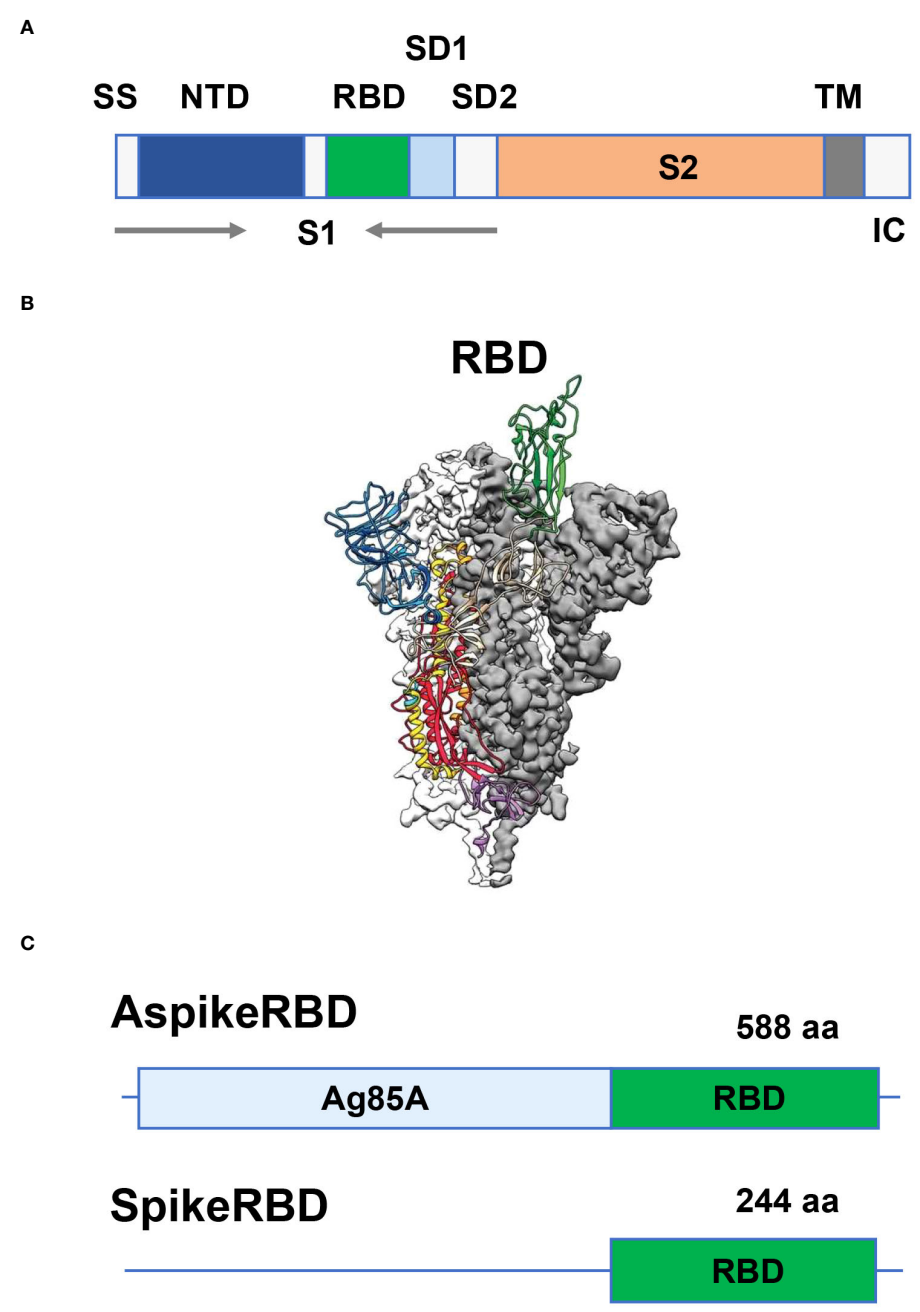
Generation of recombinant *M. bovis* BCG strains AspikeRBD and SpikeRBD. **(A)** Structure of the SARS-CoV-2 spike protein. SS, signal sequence; NTD, N-terminal domain; RBD, receptor-binding domain; SD1, subdomain 1; SD2, subdomain 2; TM, transmembrane anchor; IC, intracellular tail; S1, S1 subunit; S2, S2 subunit. **(B)** Side view of the prefusion structure of the SARS-CoV-2 spike protein with a single RBD in the up conformation (cited from Wrapp et al., 2020). **(C)** Schematic of the AspikeRBD and SpikeRBD engineering.

The genome of SARS-CoV-2 is approximately 30 kb in size, containing 14 open reading frames (ORFs) that encode 29 viral proteins. These SARS-CoV-2 proteins have been intensively studied during the development of antiviral vaccines and therapeutic drugs (Wrapp et al., 2020; Yang and Rao, 2021). Among those, four SARS-CoV-2 structural proteins, namely the nucleocapsid (N), spike (S), membrane (M) and envelope (E) proteins, draw particular attention as they are not only responsible for viral assembly but also regulate the interaction with the host, or even play a critical role in host immune evasion, modulation, and exploitation (Wrapp et al., 2020; Yang and Rao, 2021). As we described above, SARS-CoV-2 infects human cells through the recognition and binding between the RBD of its protruding S glycoprotein and human cell surface protein ACE2, which is then followed by the fusion of viral and host-cell membranes and the release of viral genomic DNA into human cells. The spike protein of SARS-CoV-2, which comprises approximately 1,200 amino acids, is a homotrimer protruding from the viral surface and can be cleaved by a furin-like protease into two functional subunits 1 (S1) and 2 (S2) in each monomer (**Figure 1A**) (Wrapp et al., 2020; Yang and Rao, 2021). As a key virulence factor in SARS-CoV-2 infection, the spike protein has become a primary focus in the development of COVID-19 vaccines and neutralizing antibodies. As shown in **Figures 1A, B**, the S1 subunit of the SARS-CoV-2 spike protein consists of two structurally independent protein domains: N-terminal domain (NTD) and C-terminal RBD. Just as its name implies, RBD is responsible for recognizing and binding the human cell surface receptor ACE2 (**Figure 1B**). Therefore, disrupting the interaction between RBD and its host receptor ACE2 becomes an effective strategy for vaccine design against COVID-19.

**Figure 2:**
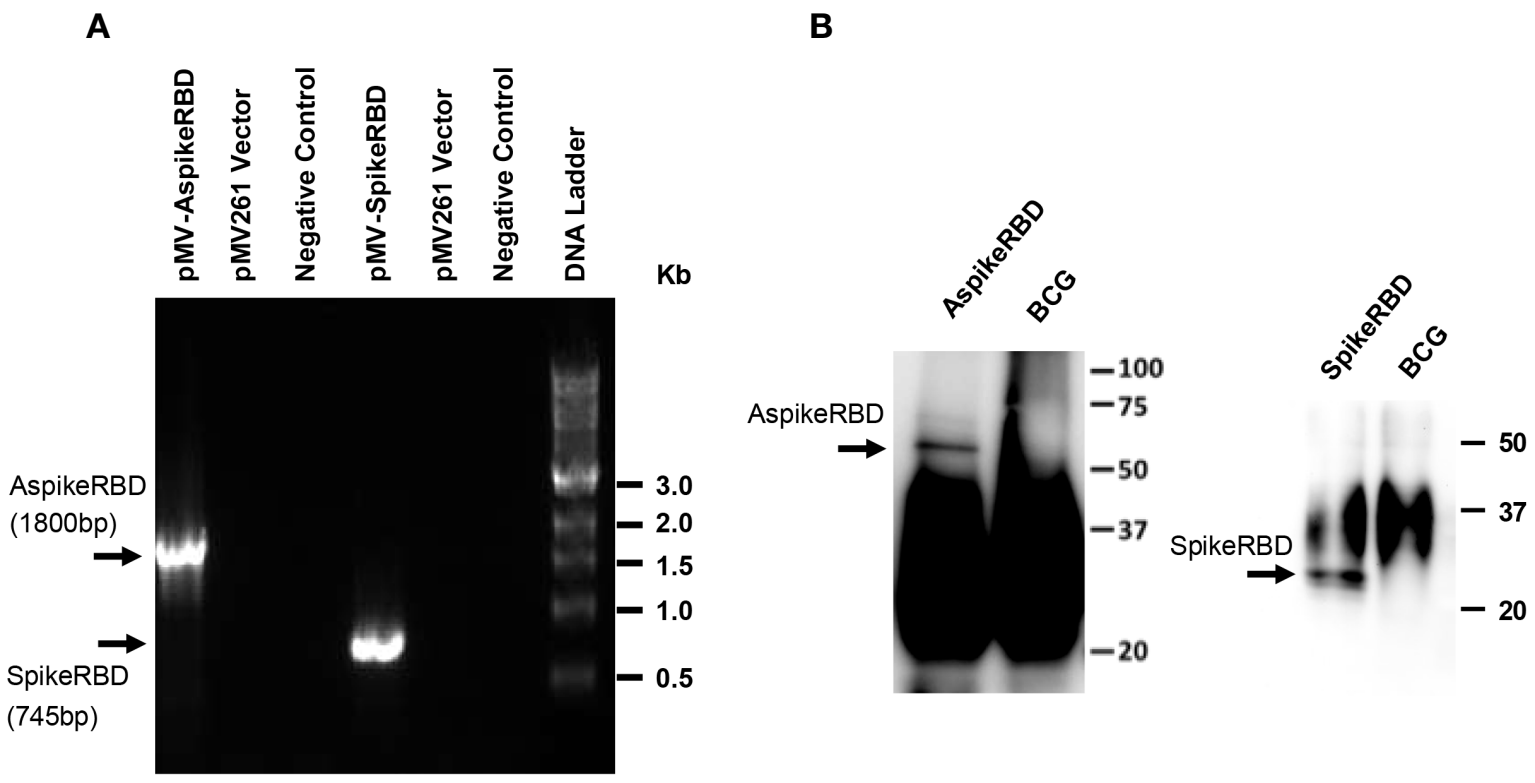
Verification of recombinant *M. bovis* BCG strains. **(A)** PCR analysis of the generated plasmids. pMV-AspikeRBD, pMV261 containing the DNA fragment encoding the fusion protein Ag85A::SpikeRBD; pMV-SpikeRBD, pMV261 containing the DNA fragment encoding the RBD domain of the SARS-CoV-2 spike protein. Negative Control, no DNA template. **(B)** Western blot for recombinant *M. bovis* BCG strains expressing AspikeRBD or SpikeRBD. Wild-type *M. bovis* BCG was used as a negative control.

In our current study, we engineered the TB vaccine strain BCG to generate a novel dual vaccine candidate against COVID-19 and TB simultaneously. As shown in **Figure 1C**, we constructed a mycobacterial plasmid constitutively expressing *M. tb* Ag85A fused with the SARS-CoV-2 spike RBD. To evaluate the potential effect of *M. tb* Ag85A on antigen immunogenicity, we also generated a control plasmid only expressing the SARS-CoV-2 spike RBD (**Figure 1C**). The generated plasmids, pMV261-Ag85A::SpikeRBD (AspikeRBD) and pMV261-SpikeRBD, were verified by PCR (**Figure 2A**, **Table 1**) and further confirmed by DNA sequencing analysis. Finally, the two plasmids were then transformed into the wild-type BCG strain respectively, and the expression of the AspikeRBD and SpikeRBD proteins in recombinant BCG strains was validated by Western blot (**Figure 2B**).

### *M. bovis* BCG AspikeRBD induced SARS-CoV-2 spike-specific immune responses in mice

**Figure 3:**
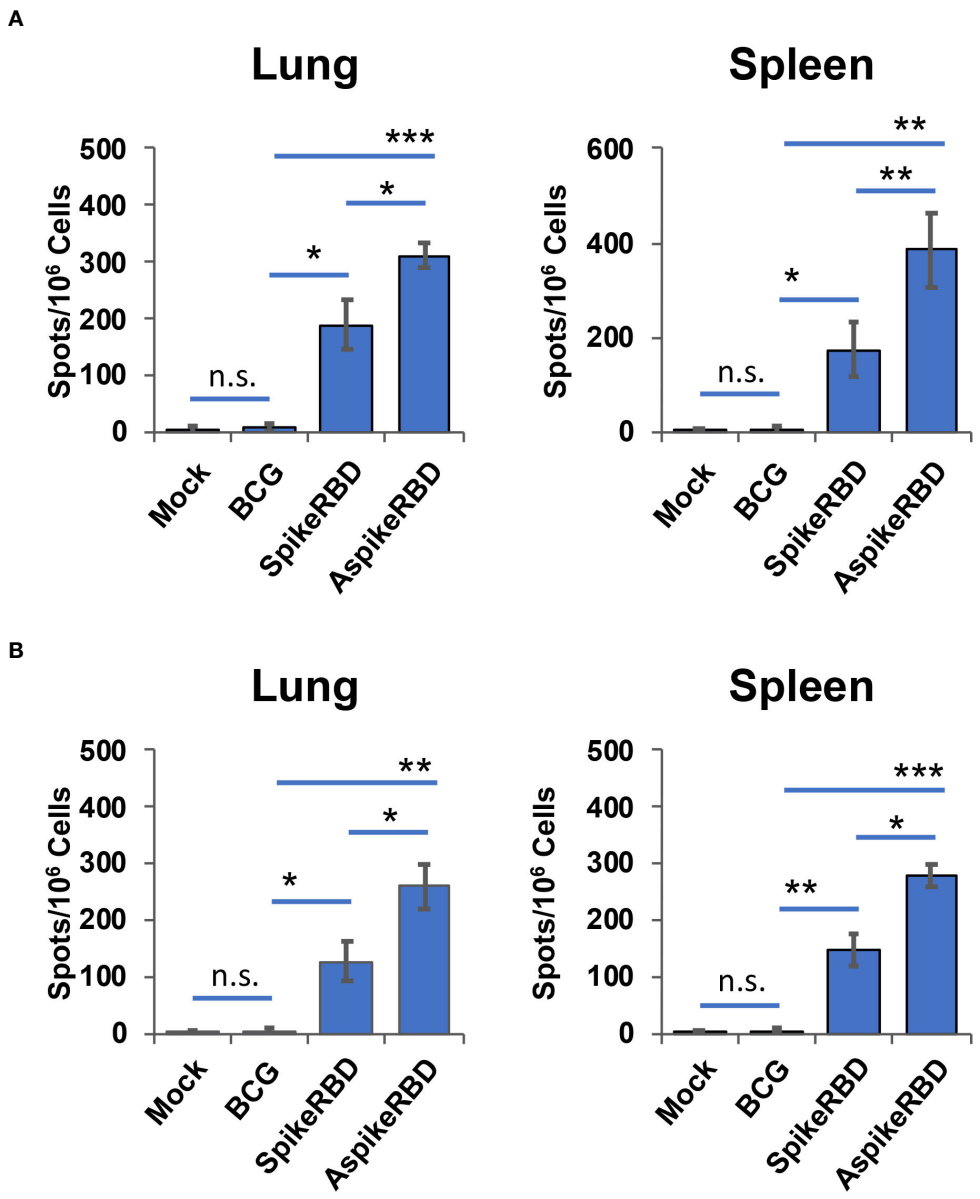
Spike-specific T-cell response in C57BL/6 mice immunized with recombinant *M. bovis* BCG strains. The number of antigen-specific IFNγ-releasing T-cells in the lung and spleen was determined by ELISPOT analysis in the presence of the recombinant SARS-CoV-2 spike full protein **(A)** or RBD **(B)**. Data are mean ± SD (n=3/group) and representatives of two independent experiments. Mock, unimmunized. n.s., not statistically significant; *p < 0.05, **p < 0.01 and ***p < 0.001 by two-tailed Student’s t-test.

An effective vaccine against COVID-19 requires both neutralizing antibodies and a Th1-driven cellular component (Ewer et al., 2021). To determine the immunogenicity of our recombinant BCG strains in preclinical animal models, we subcutaneously immunized wild-type C57BL/6 mice as described in Materials and Methods. T-cell activation in the lung and spleen, and SARS-CoV-2 spike RBD-specific IgG production in the serum were evaluated 4 weeks post-immunization. To understand the efficacy of recombinant BCG strains in stimulating spike-specific T-cell activation, we treated the single-cell suspension of mouse lung and spleen *ex vivo* using recombinant SARS-CoV-2 spike full protein (**Figure 3A**) or RBD (**Figure 3B**). As shown in **Figure 3**, compared to parental BCG-immunized or unimmunized mice, either recombinant spike full protein or RBD significantly induced T-cell activation and IFNγ-release in the lung and spleen cells prepared from mice immunized with AspikeRBD- or SpikeRBD-expressing recombinant BCG strain. It is notable that immunization with *M. bovis* BCG AspikeRBD that expresses the fusion protein AspikeRBD, rather than with *M. bovis* BCG SpikeRBD that expresses the spike RBD alone, generated much stronger T-cell activation and IFNγ-release in the lung and spleen cell culture in the presence of the spike full protein (**Figure 3A**) or RBD (**Figure 3B**).

**Figure 4:**
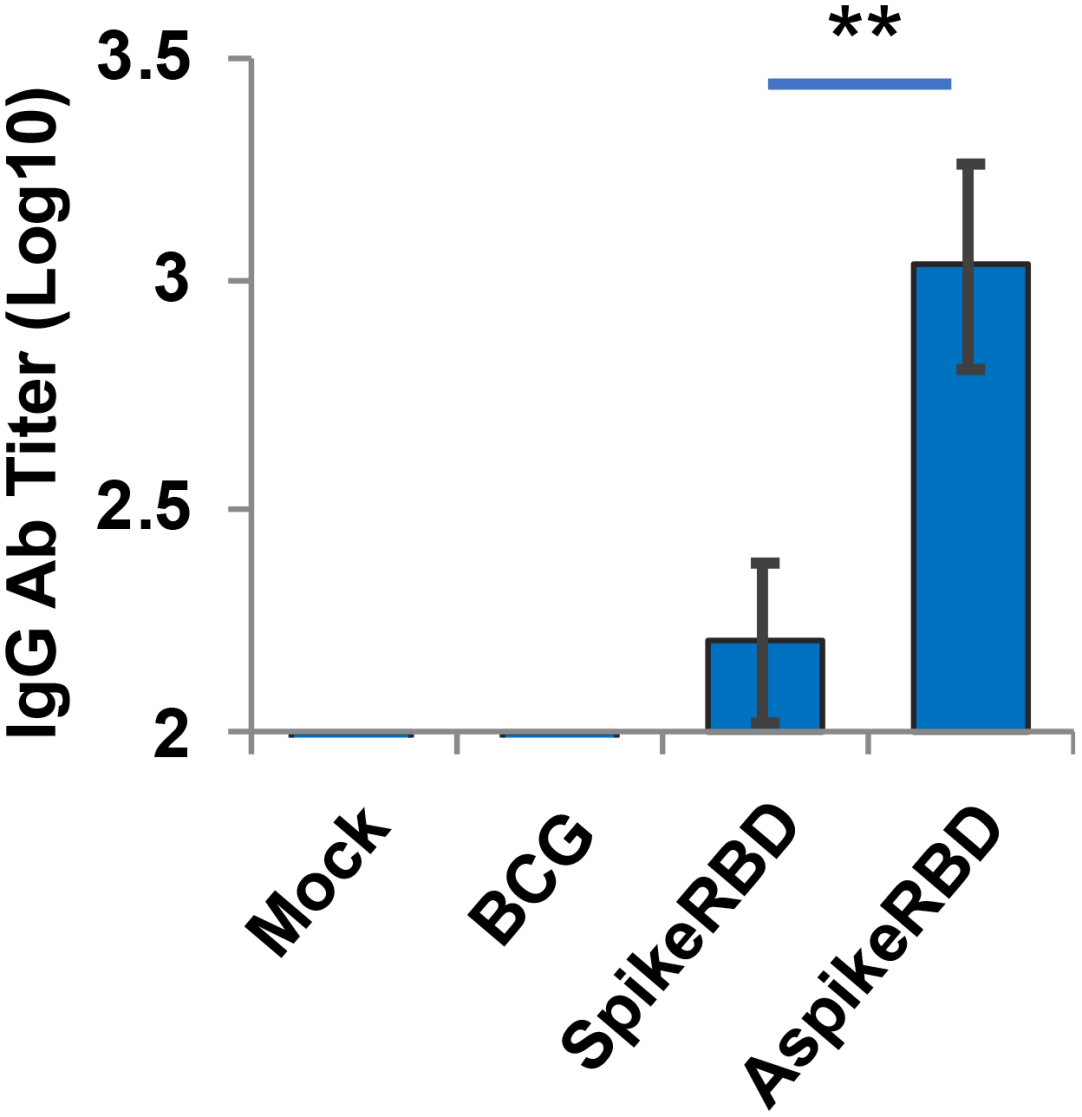
Titration of spike RBD-specific IgG in C57BL/6 mice immunized with recombinant *M. bovis* BCG strains. SARS-CoV-2 spike RBD-specific IgG endpoint titers were determined using mouse serum that was collected 4 weeks after *M.bovis* BCG immunization. Mean reciprocal dilutions are used as the endpoint titer (log10). Data shown are the mean ± SD (n=3/group) and representative of two independent experiments. Mock, unimmunized. **p < 0.01 by two-tailed Student’s t-test.

As we described above, the SARS-CoV-2 spike protein recognizes and binds to human ACE2 receptor via its RBD. Therefore, the capability to produce RBD-neutralizing antibodies has been used as one of the criteria to evaluate the efficacy of COVID-19 vaccines (Barouch, 2022). In this study, we analyzed the titration of IgG antibodies that specifically recognized SARS-CoV-2 spike RBD in the serum 4 weeks post-immunization. As shown in **Figure 4**, recombinant BCG strains expressing AspikeRBD or SpikeRBD, but not the parental BCG strain, significantly induced the production of IgG antibodies specific for SARS-CoV-2 spike RBD. Furthermore, coincident with T-cell activation (**Figure 3**), mice immunized with *M. bovis* BCG expressing AspikeRBD produced a higher level of RBD-specific IgG antibodies in the serum 4 weeks post-immunization compared to those immunized with *M. bovis* BCG SpikeRBD expressing the spike RBD alone.

### *M. bovis* BCG AspikeRBD increased *M. tb* Ag85A-specific immune response in mice

**Figure 5:**
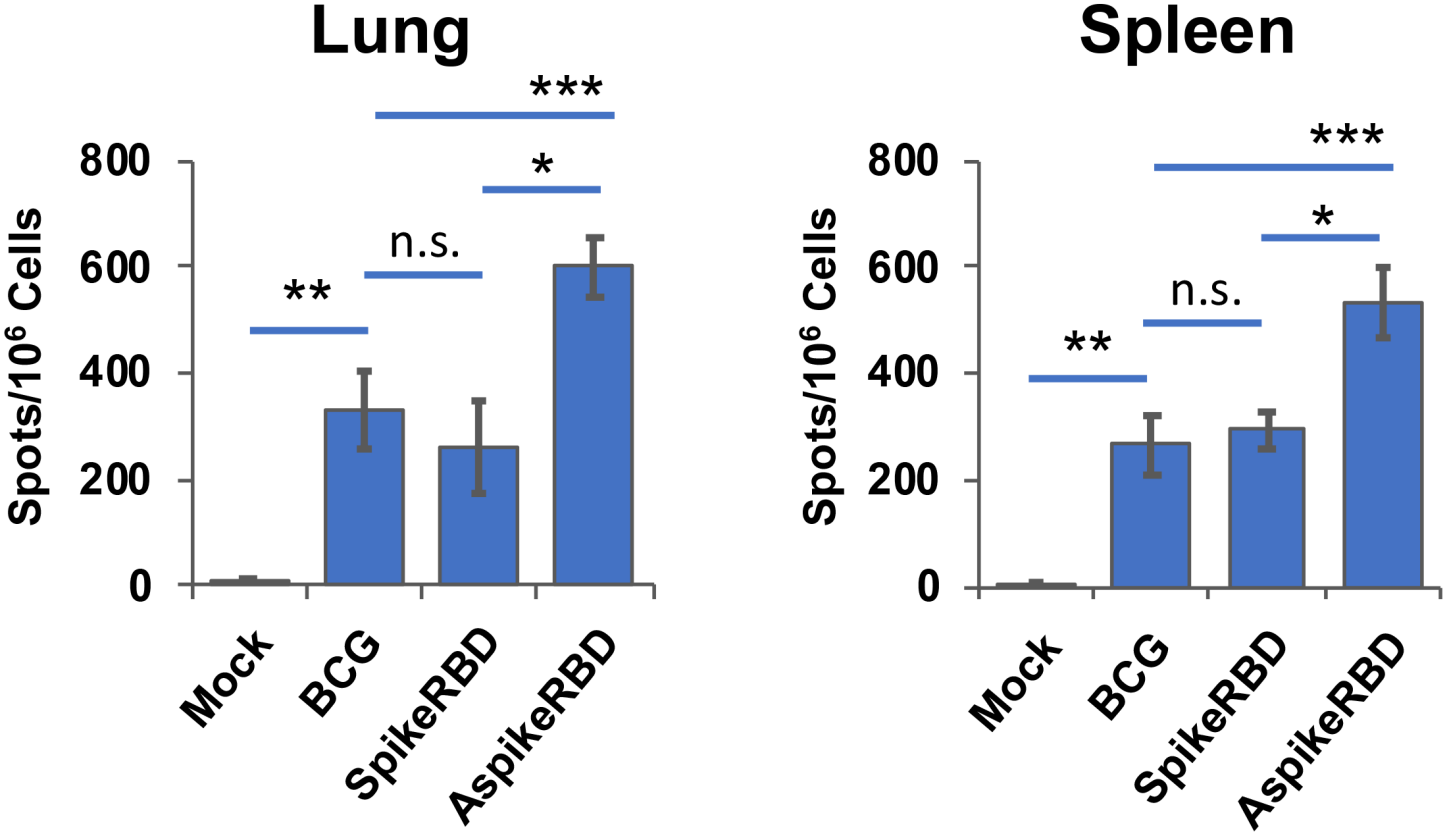
*M. tb* Ag85A-specific T-cell response in C57BL/6 mice immunized with recombinant *M. bovis* BCG strains. The number of antigen-specific IFNγ-releasing T-cells in the lung and spleen was determined by ELISPOT analysis in the presence of the *M. tb* Ag85A protein. Data are mean ± SD (n=3/group) and representatives of two independent experiments. Mock, unimmunized. n.s., not statistically significant; *p < 0.05, **p < 0.01 and ***p < 0.001 by two-tailed Student’s t test.

In the context of TB immunization, recombinant BCG strains expressing *M. tb* protein antigens have been widely studied, including the recombinant BCG strain overexpressing *M. tb* Ag85A. It has been found that the recombinant BCG strain overexpressing *M. tb* Ag85A induced more *M. tb* Ag85A-specific immune responses and protected experimental guinea pigs and monkeys from *M. tb* lung infection better than parental BCG (Sugawara et al., 2007a; Sugawara et al., 2007b; Sugawara et al., 2009). In our current study, we also evaluated *M. tb* Ag85A-specfic immune response in mice immunized with recombinant BCG strains expressing AspikeRBD or SpikeRBD. Th1 response is critical for protection against *M. tb* and has been widely used as a standard to evaluate novel TB vaccine candidates (Flynn and Chan, 2001). As shown in **Figure 5**, compared to unimmunized mice, all BCG strains drastically increased the number of *M. tb* Ag85A-specific IFNγ-releasing T cells in mouse lung and spleen, which is expected since *M. tb* and BCG Ag85A proteins share very high sequence homology. It is worthy of attention that, among these BCG strains, *M. bovis* BCG AspikeRBD, which constitutively expresses the fusion protein AspikeRBD, induced much stronger *M. tb* Ag85A-specific T-cell activation and IFNγ-release in mouse lung and spleen when compared to parental BCG strain or spikeRBD strain.

**Figure 6:**
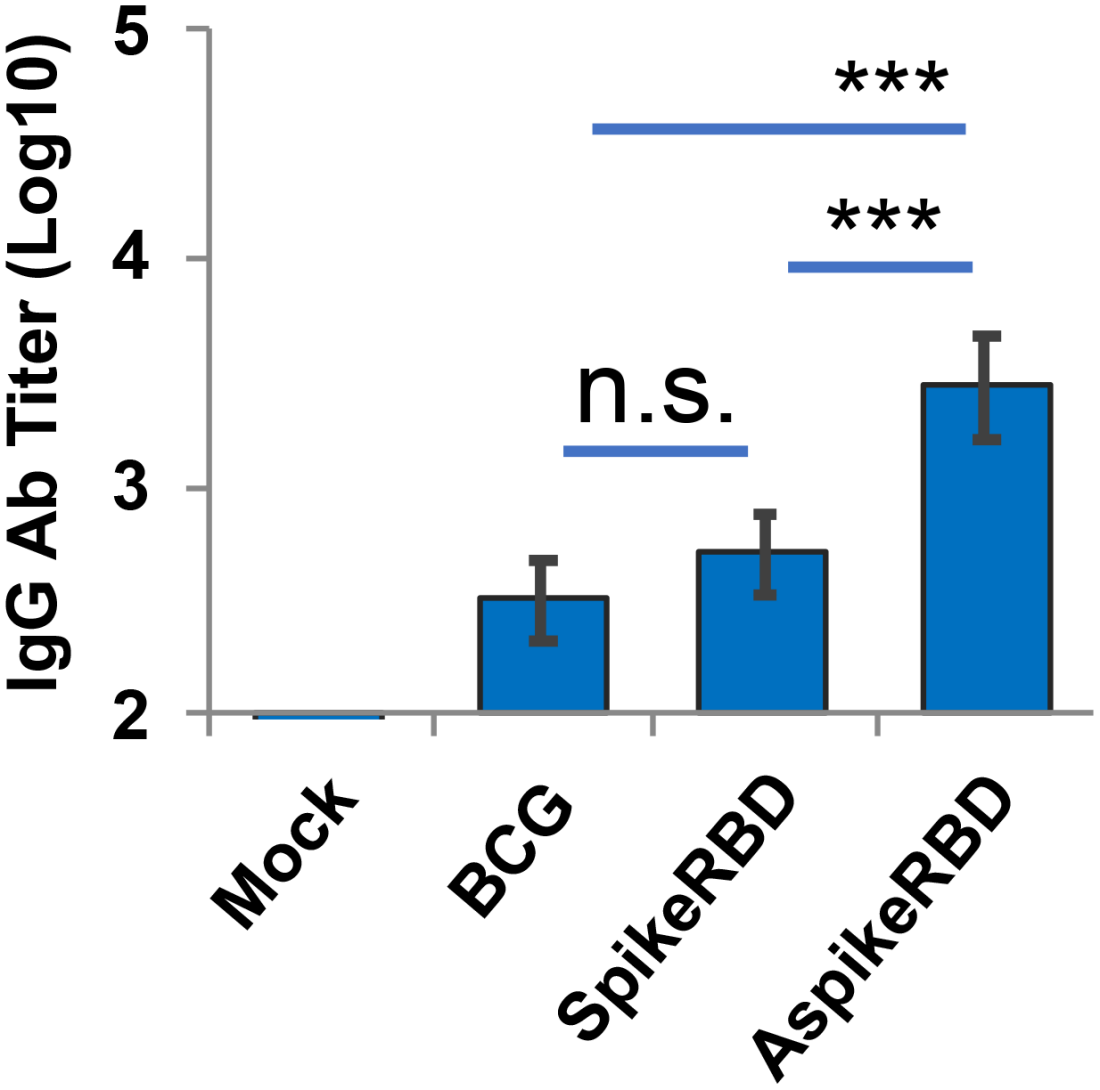
Titration of *M. tb* Ag85A-specific IgG in C57BL/6 mice immunized with recombinant *M. bovis* BCG strains. *M. tb* Ag85A-specific IgG endpoint titers were determined using mouse serum that was collected 4 weeks after *M.bovis* BCG immunization. Mean reciprocal dilutions are used as the endpoint titer (log10). Data shown are the mean ± SD (n=3/group) and representative of two independent experiments. Mock, unimmunized. n.s., not statistically significant; ***p < 0.001 by two-tailed Student’s t test.

Consistent with the T-cell activation (**Figure 5**), all BCG strains induced the production of *M. tb* Ag85A-specific IgG antibodies as well compared to the unimmunized group in mice (**Figure 6**). Once more, mice immunized with *M. bovis* BCG AspikeRBD showed a remarkably elevated level of *M. tb* Ag85A-specific IgG in the serum compared to those immunized with parental BCG strain or spikeRBD strain (**Figure 6**). As shown in **Figures 5** and **6**, the expression of SpikeRBD alone in BCG had no effect on the activation of *M. tb* Ag85A-specific IFNγ-releasing T-cells (**Figure 5**) in the lung and spleen, and the production of *M. tb* Ag85A-specific IgG antibodies (**Figure 6**) in the serum in mice.

## Discussion

In the past decades, researchers have made considerable effort on the innovative application of live attenuated bacteria as vaccine vectors against a variety of human diseases from viral infections to different cancers. Recombinant bacterial strains expressing tumor antigens have been studied as novel vaccine candidates against tumors and displayed promising activity against the progression of various tumors in experimental animal models. Among these tumor vaccine candidates, a gram-positive facultative intracellular pathogen that can actively invade and replicate within human mononuclear cells, *Listeria monocytogenes*, draws a lot of attention as an effective vaccine platform since it can be genetically engineered to express and deliver human tumor antigens. As a promising bacterial vector, bioengineered live attenuated *L. monocytogenes* is able to trigger robust T-cell immune responses against tumor-specific antigens and exhibits great potential in vaccine development. For example, an attenuated *L. monocytogenes* was genetically modified to express the tumor antigen Kras^G12D^, and significantly inhibited the progression of pancreatic ductal adenocarcinoma (PDA) in mouse model (Keenan et al., 2014). Another *L. monocytogenes*-based vaccine candidate, Lmdd (LM ΔdalΔdat)-MPFG (multiple peptide fusing genes), was developed to express and secrete hepatocellular carcinoma (HCC)-related tumor-associated antigens, thereby triggering anti-tumor immune responses *in vitro* and in mouse model (Chen et al., 2012). For the immunotherapy of prostate cancer (PCa), the second most common cause of cancer death among men in the United States, a recombinant live attenuated *L. monocytogenes* strain (Lm–LLO–PSA) expressing human prostate-specific antigen (PSA) was found to elicit strong immune responses toward PSA and regress established tumors in a murine model, indicating its potential application as a therapeutic vaccine (Shahabi et al., 2008). Besides delivering human antigens, *L. monocytogenes* has also been engineered to express viral proteins related to human cancers. For example, two recombinant *L. monocytogenes* strains expressing human papilloma virus-16 (HPV-16) E7 significantly induced antigen-specific T-cell activation and regressed established syngeneic tumors immortalized by HPV-16 in mouse model (Gunn et al., 2001). Consistent results have been observed with a recombinant *L. monocytogenes* strain expressing influenza nucleoprotein (NP) (Ikonomidis et al., 1994).

Similar to *L. monocytogenes*, BCG has the capacity to actively enter, replicate and proliferate within mononuclear cells, particularly macrophages. Thus, the antigens carried by an attenuated BCG-based vector will be conveniently recognized by macrophages, and then swiftly stimulate antigen-specific defensive immune responses within the host, including both innate immune responses and adaptive immune responses that to a great extent shaped by the former. Moreover, BCG has been used as a human vaccine for over a century and is considered to be the most widely used vaccine worldwide (WHO, 2021), which makes it a safer and better option for the vaccine platform. Besides TB, BCG vaccination has been proved to be able to trigger non-specific protective effects against respiratory tract infections caused by other microbial pathogens, including viruses and bacteria (Aaby et al., 2011; Moorlag et al., 2019; Giamarellos-Bourboulis et al., 2020; Gonzalez-Perez et al., 2021; Li et al., 2021). Recent studies suggest that BCG vaccination provides protection against the morbidity, severity, hospitalization, and mortality of COVID-19 (Berg et al., 2020; Escobar et al., 2020; Moorlag et al., 2020; Weng and Chan, 2020; Rivas et al., 2021; Hilligan et al., 2022). Clinical trials also proved that priming by BCG markedly enhanced the capacity of its following vaccination to induce antigen-specific antibody responses against the pathogens (Leentjens et al., 2015; Arts et al., 2018). In the field of cancer, the concept of using BCG as an immunotherapy against cancer was pioneered by Raymond Pearl in 1929. Subsequently, this anti-neoplastic feature of BCG was confirmed in animal models (Old et al., 1959; Zbar and Tanaka, 1971; Zbar and Rapp, 1974). A growing body of clinical evidence indicates that BCG can stimulate the anti-cancer immunity, inhibit cancer progression and metastases, and reduce relapse rates, which prompts the application of BCG in cancer immunotherapy (Mathé et al., 1969; Morton et al., 1974). Clinical data from a 60-year follow-up trial demonstrated that BCG-vaccinated children were less susceptible to lung cancer (Usher et al., 2019). In bladder cancer treatment, BCG has been established as an anti-cancer immunotherapy due to its excellent performance in preventing recurrence and progression (Sylvester et al., 2002; Shelley et al., 2004; Babjuk et al., 2022). Although the precise mechanism of action by which BCG exerts its immune-enhancing effect remains ambiguous, it is intriguing to harness this immune power for the development of robust novel vaccines. Different from conventional vaccines containing killed or live attenuated pathogens, which possess inherent immune-potentiating activity, modern vaccines are usually highly purified proteins or peptides with poor immunogenicity due to their small size and/or the removal of virulence factors. Therefore, a safe and effective immune stimulant (or adjuvant), like BCG, will greatly enhance the vaccine performance. So far, researchers have already turned their attention to transforming this classical TB vaccine into a potent self-adjuvant vaccine platform helping fight against various human infectious diseases other than TB (Mouhoub et al., 2021).

Four COVID-19 vaccines are currently authorized by the United States Food and Drug Administration (FDA), including three major types based on the vaccine platform: messenger RNA (mRNA), viral vector, and adjuvanted protein subunit vaccines. First, mRNA vaccines from Pfizer-BioNTech and Moderna are delivered by lipid nanoparticles (LNPs). After vaccination, the mRNA will be internalized and guided into the cytosol but not the nucleus of the transfected human cells, where the SARS-CoV-2-specific antigens will be produced and then presented onto the cell surface. As a result, these SARS-CoV-2-specific antigens will be recognized by the human immune system and induce potent SARS-CoV-2-specific cellular and humoral immune responses, including T-cell activation and neutralizing antibody production (Hogan and Pardi, 2022). Second, the virus-vectored COVID-19 vaccine from Johnson & Johnson’s Janssen (J&J/Janssen) uses a recombinant, replication-incompetent adenovirus serotype 26 (Ad26) platform expressing a modified full-length SARS-CoV-2 spike protein. After vaccination, once human cells display modified spike protein on their surface, the host immune system is activated to produce SARS-CoV-2-specific neutralizing antibodies and defensive lymphocytes (Sadoff et al., 2021; Barouch, 2022). Third, Novavax protein subunit COVID-19 vaccine contains a recombinant SARS-CoV-2 spike protein expressed via insect cells *Spodoptera frugiperda* Sf9 and requires a plant (*Quillaja saponaria*)-derived immuno-adjuvant Matrix-M in the formulation (Bengtsson et al., 2016; Hotez and Bottazzi, 2022). Successfully delivered spike protein can be detected immediately by the human immune system after vaccination. Then, neutralizing antibodies and defensive immunity will be induced to protect the recipients against COVID-19 (Barouch, 2022). In our current study, we generated a recombinant BCG strain, *M. bovis* BCG AspikeRBD, and confirmed that it expressed a fusion protein composed of the *M. tb* Ag85A protein and the SARS-CoV-2 spike RBD domain as expected while it remains unclear if the RBD is exposed outward as it in the SARS-CoV-2 spike protein. Our results demonstrated that *M. bovis* BCG AspikeRBD induced robust protective immune responses in mice, including spike-specific T-cell activation and effector cytokine release, as well as IgG production, which represent hallmarks of an effective vaccine against COVID-19. Furthermore, compared to the parental BCG strain, *M. bovis* BCG AspikeRBD elicited much stronger *M. tb*-specific immune response in mice.

We have recognized the limitations within this study: 1) We only measured the immune response at 4 weeks after BCG vaccination. Multiple time points such as 2, 4, 8 and 12 weeks would have improved the rigor of the study. Since this was the first study investigating the potential application of a recombinant BCG strain expressing a fusion protein consisting of an *M. tb* antigen and a truncated SARS-CoV-2 protein as a novel dual vaccine, we verified it at a time point that has been widely used in the development of BCG-based vaccines against *M. tb*. In the future study, we will further evaluate the efficacy of *M. bovis* BCG AspikeRBD in mice at multiple time points after vaccination. Additionally, we will also determine SARS-CoV-2 and *M. tb* infection in vaccinated mice. 2) In this study, mice were only vaccinated with BCG strains once. Considering the significant effect of a boost vaccination in the prevention of COVID-19, a boost vaccination with *M. bovis* BCG AspikeRBD would potentially improve the efficacy of vaccination. In the future study, we will further test *M. bovis* BCG AspikeRBD with primary and booster vaccination in mice. 3) The BCG vaccine for TB is freeze-dried before use. While the recombinant BCG strains in this study were stored at − 80°C until use, it would be worth to evaluate our recombinant BCG strains after freeze-drying.

Taken together, our results indicate that the immuno-stimulating activity of BCG can be harnessed to develop a potent self-adjuvant vaccine platform that expresses and displays SARS-CoV-2- and *M. tb*-specific antigens simultaneously, improves vaccine immunogenicity, facilitates antigen-presentation, and ultimately enhances protective immune responses against COVID-19 and TB. Based on this effective dual vaccine platform, antigenic variability of SARS-CoV-2 can be overcome by the expression of an updated fusion protein containing the new virus-specific antigen in a similar way as the mRNA vaccine. Meanwhile, our dual vaccine candidate is more convenient to transport and store than the current mRNA vaccines. Therefore, our dual vaccine candidate *M. bovis* BCG AspikeRBD possesses unique immune and practical features, and we believe these features make it a promising candidate to help prevent future outbreaks of COVID-19 and TB.

## Data availability statement

The raw data supporting the conclusions of this article will be made available by the authors, without undue reservation.

## Ethics statement

The animal study was approved by The Institutional Animal Care and Use Committee (IACUC) of Oklahoma State University. The study was conducted in accordance with the local legislation and institutional requirements.

## Author contributions

CG: Conceptualization, Methodology, Data curation, Investigation, Writing – review and editing. XT: Conceptualization, Methodology, Writing – review and editing. AM: Investigation, Methodology, Writing – review and editing. AS: Investigation, Methodology, Writing – review and editing. LL: Funding acquisition, Resources, Writing – review and editing. YC: Funding acquisition, Resources, Conceptualization, Investigation, Methodology, Supervision, Writing – original draft.

## References

[B1] Aaby P. Roth A. Ravn H. Napirna B. M. Rodrigues A. Lisse I. M.. (2011). Randomized trial of BCG vaccination at birth to low-birth-weight children: beneficial nonspecific effects in the neonatal period? J. Infect. Dis. 204 (2), 245–252. doi: 10.1093/infdis/jir240 21673035

[B2] Andersen P. Scriba T. J. (2019). Moving tuberculosis vaccines from theory to practice. Nat. Rev. Immunol. 19 (9), 550–562. doi: 10.1038/s41577-019-0174-z 31114037

[B3] Arts R. J. W. Moorlag S. Novakovic B. Li Y. Wang S. Y. Oosting M.. (2018). BCG Vaccination Protects against Experimental Viral Infection in Humans through the Induction of Cytokines Associated with Trained Immunity. Cell Host Microbe 23 (1), 89–100.e105. doi: 10.1016/j.chom.2017.12.010 29324233

[B4] Babjuk M. Burger M. Capoun O. Cohen D. Compérat E. M. Dominguez Escrig J. L.. (2022). European association of urology guidelines on non-muscle-invasive bladder cancer (Ta, T1, and carcinoma in situ). Eur. Urol 81 (1), 75–94. doi: 10.1016/j.eururo.2021.08.010 34511303

[B5] Barouch D. H. (2022). Covid-19 vaccines - immunity, variants, boosters. N Engl. J. Med. 387 (11), 1011–1020. doi: 10.1056/NEJMra2206573 36044620 PMC9454645

[B6] Bengtsson K. L. Song H. Stertman L. Liu Y. Flyer D. C. Massare M. J.. (2016). Matrix-M adjuvant enhances antibody, cellular and protective immune responses of a Zaire Ebola/Makona virus glycoprotein (GP) nanoparticle vaccine in mice. Vaccine 34 (16), 1927–1935. doi: 10.1016/j.vaccine.2016.02.033 26921779

[B7] Berg M. K. Yu Q. Salvador C. E. Melani I. Kitayama S. (2020). Mandated Bacillus Calmette-Guérin (BCG) vaccination predicts flattened curves for the spread of COVID-19. Sci. Adv. 6 (32), eabc1463. doi: 10.1126/sciadv.abc1463 32923613 PMC7457335

[B8] Chapman R. Chege G. Shephard E. Stutz H. Williamson A. L. (2010). Recombinant Mycobacterium bovis BCG as an HIV vaccine vector. Curr. HIV Res. 8 (4), 282–298. doi: 10.2174/157016210791208686 20353397 PMC3188323

[B9] Chauchet X. Hannani D. Djebali S. Laurin D. Polack B. Marvel J.. (2016). Poly-functional and long-lasting anticancer immune response elicited by a safe attenuated Pseudomonas aeruginosa vector for antigens delivery. Mol. Ther. Oncolytics 3, 16033. doi: 10.1038/mto.2016.33 28035332 PMC5155632

[B10] Chen Y. Yang D. Li S. Gao Y. Jiang R. Deng L.. (2012). Development of a Listeria monocytogenes-based vaccine against hepatocellular carcinoma. Oncogene 31 (17), 2140–2152. doi: 10.1038/onc.2011.395 21927025

[B11] Cheng Y. Kiene N. J. Tatarian A. Eix E. F. Schorey J. S. (2020). Host cytosolic RNA sensing pathway promotes T Lymphocyte-mediated mycobacterial killing in macrophages. PloS Pathog. 16 (5), e1008569. doi: 10.1371/journal.ppat.1008569 32463840 PMC7282665

[B12] Cheng Y. Schorey J. S. (2013). Exosomes carrying mycobacterial antigens can protect mice against Mycobacterium tuberculosis infection. Eur. J. Immunol. 43 (12), 3279–3290. doi: 10.1002/eji.201343727 23943377 PMC4076847

[B13] Cheng Y. Schorey J. S. (2016). Targeting soluble proteins to exosomes using a ubiquitin tag. Biotechnol. Bioengineering 113 (6), 1315–1324. doi: 10.1002/bit.25884 26574179

[B14] Cheng Y. Schorey J. S. (2018). Mycobacterium tuberculosis–induced IFN-β production requires cytosolic DNA and RNA sensing pathways. J. Exp. Med. 215 (11), 2919–2935. doi: 10.1084/jem.20180508 30337468 PMC6219742

[B15] Cheng Y. Schorey J. S. (2019). Extracellular vesicles deliver Mycobacterium RNA to promote host immunity and bacterial killing. EMBO Rep. 20 (3), e46613. doi: 10.15252/embr.201846613 30683680 PMC6399609

[B16] Dennehy M. Bourn W. Steele D. Williamson A. L. (2007). Evaluation of recombinant BCG expressing rotavirus VP6 as an anti-rotavirus vaccine. Vaccine 25 (18), 3646–3657. doi: 10.1016/j.vaccine.2007.01.087 17339069

[B17] Edwards A. M. Baric R. S. Saphire E. O. Ulmer J. B. (2022). Stopping pandemics before they start: Lessons learned from SARS-CoV-2. Science 375 (6585), 1133–1139. doi: 10.1126/science.abn1900 35271333

[B18] Escobar L. E. Molina-Cruz A. Barillas-Mury C. (2020). BCG vaccine protection from severe coronavirus disease 2019 (COVID-19). Proc. Natl. Acad. Sci. U.S.A. 117 (30), 17720–17726. doi: 10.1073/pnas.2008410117 32647056 PMC7395502

[B19] Ewer K. J. Barrett J. R. Belij-Rammerstorfer S. Sharpe H. Makinson R. Morter R.. (2021). T cell and antibody responses induced by a single dose of ChAdOx1 nCoV-19 (AZD1222) vaccine in a phase 1/2 clinical trial. Nat. Med. 27 (2), 270–278. doi: 10.1038/s41591-020-01194-5 33335323

[B20] Flynn J. L. Chan J. (2001). Immunology of tuberculosis. Annu. Rev. Immunol. 19, 93–129. doi: 10.1146/annurev.immunol.19.1.93 11244032

[B21] Giamarellos-Bourboulis E. J. Tsilika M. Moorlag S. Antonakos N. Kotsaki A. Domínguez-Andrés J.. (2020). Activate: randomized clinical trial of BCG vaccination against infection in the elderly. Cell 183 (2), 315–323.e319. doi: 10.1016/j.cell.2020.08.051 32941801 PMC7462457

[B22] Gonzalez-Perez M. Sanchez-Tarjuelo R. Shor B. Nistal-Villan E. Ochando J. (2021). The BCG vaccine for COVID-19: first verdict and future directions. Front. Immunol. 12. doi: 10.3389/fimmu.2021.632478 PMC7982405 33763077

[B23] Gunn G. R. Zubair A. Peters C. Pan Z. K. Wu T. C. Paterson Y. (2001). Two Listeria monocytogenes vaccine vectors that express different molecular forms of human papilloma virus-16 (HPV-16) E7 induce qualitatively different T cell immunity that correlates with their ability to induce regression of established tumors immortalized by HPV-16. J. Immunol. 167 (11), 6471–6479. doi: 10.4049/jimmunol.167.11.6471 11714814

[B24] Heinz F. X. Stiasny K. (2021). Distinguishing features of current COVID-19 vaccines: knowns and unknowns of antigen presentation and modes of action. NPJ Vaccines 6, 104. doi: 10.1038/s41541-021-00369-6 34400651 PMC8368295

[B25] Hilligan K. L. Namasivayam S. Clancy C. S. O’Mard D. Oland S. D. Robertson S. J.. (2022). Intravenous administration of BCG protects mice against lethal SARS-CoV-2 challenge. J. Exp. Med. 219 (2):e20211862. doi: 10.1084/jem.20211862 34889942 PMC8669500

[B26] Hogan M. J. Pardi N. (2022). mRNA vaccines in the COVID-19 pandemic and beyond. Annu. Rev. Med. 73, 17–39. doi: 10.1146/annurev-med-042420-112725 34669432

[B27] Hotez P. J. Bottazzi M. E. (2022). Whole inactivated virus and protein-based COVID-19 vaccines. Annu. Rev. Med. 73 (1), 55–64. doi: 10.1146/annurev-med-042420-113212 34637324

[B28] Ikonomidis G. Paterson Y. Kos F. J. Portnoy D. A. (1994). Delivery of a viral antigen to the class I processing and presentation pathway by Listeria monocytogenes. J. Exp. Med. 180 (6), 2209–2218. doi: 10.1084/jem.180.6.2209 7964496 PMC2191788

[B29] Jia Q. Bielefeldt-Ohmann H. Maison R. M. Masleša-Galić S. Cooper S. K. Bowen R. A.. (2021). Replicating bacterium-vectored vaccine expressing SARS-CoV-2 Membrane and Nucleocapsid proteins protects against severe COVID-19-like disease in hamsters. NPJ Vaccines 6 (1), 47. doi: 10.1038/s41541-021-00321-8 33785745 PMC8009914

[B30] Keenan B. P. Saenger Y. Kafrouni M. I. Leubner A. Lauer P. Maitra A.. (2014). A Listeria vaccine and depletion of T-regulatory cells activate immunity against early stage pancreatic intraepithelial neoplasms and prolong survival of mice. Gastroenterology 146 (7), 1784–1794.e1786. doi: 10.1053/j.gastro.2014.02.055 24607504 PMC4035450

[B31] Kilpeläinen A. Saubi N. Guitart N. Moyo N. Wee E. G. Ravi K.. (2019). Priming with recombinant BCG expressing novel HIV-1 conserved mosaic immunogens and boosting with recombinant chAdOx1 is safe, stable, and elicits HIV-1-specific T-cell responses in BALB/c mice. Front. Immunol. 10. doi: 10.3389/fimmu.2019.00923 PMC6530512 31156614

[B32] Kim B. J. Kim B. R. Kook Y. H. Kim B. J. (2018). Development of a live recombinant BCG expressing human immunodeficiency virus type 1 (HIV-1) gag using a pMyong2 vector system: potential use as a novel HIV-1 vaccine. Front. Immunol. 9. doi: 10.3389/fimmu.2018.00643 PMC5880907 29636755

[B33] Leentjens J. Kox M. Stokman R. Gerretsen J. Diavatopoulos D. A. van Crevel R.. (2015). BCG vaccination enhances the immunogenicity of subsequent influenza vaccination in healthy volunteers: A randomized, placebo-controlled pilot study. J. Infect. Dis. 212 (12), 1930–1938. doi: 10.1093/infdis/jiv332 26071565

[B34] Li C. Wen A. Shen B. Lu J. Huang Y. Chang Y. (2011). FastCloning: a highly simplified, purification-free, sequence- and ligation-independent PCR cloning method. BMC Biotechnol. 11, 92. doi: 10.1186/1472-6750-11-92 21992524 PMC3207894

[B35] Li J. Zhan L. Qin C. (2021). The double-sided effects of Mycobacterium Bovis bacillus Calmette–Guérin vaccine. NPJ Vaccines 6 (1), 14. doi: 10.1038/s41541-020-00278-0 33495451 PMC7835355

[B36] Lin I. Y. Van T. T. Smooker P. M. (2015). Live-attenuated bacterial vectors: tools for vaccine and therapeutic agent delivery. Vaccines (Basel) 3 (4), 940–972. doi: 10.3390/vaccines3040940 26569321 PMC4693226

[B37] Mathé G. Amiel J. L. Schwarzenberg L. Schneider M. Cattan A. Schlumberger J. R.. (1969). Active immunotherapy for acute lymphoblastic leukæmia. Lancet 293 (7597), 697–699. doi: 10.1016/S0140-6736(69)92648-8 4182654

[B38] Matsumoto S. Yukitake H. Kanbara H. Yamada T. (1998). Recombinant Mycobacterium bovis bacillus Calmette-Guérin secreting merozoite surface protein 1 (MSP1) induces protection against rodent malaria parasite infection depending on MSP1-stimulated interferon gamma and parasite-specific antibodies. J. Exp. Med. 188 (5), 845–854. doi: 10.1084/jem.188.5.845 9730886 PMC2213399

[B39] Moorlag S. Arts R. J. W. van Crevel R. Netea M. G. (2019). Non-specific effects of BCG vaccine on viral infections. Clin. Microbiol. Infect. 25 (12), 1473–1478. doi: 10.1016/j.cmi.2019.04.020 31055165

[B40] Moorlag S. van Deuren R. C. van Werkhoven C. H. Jaeger M. Debisarun P. Taks E.. (2020). Safety and COVID-19 symptoms in individuals recently vaccinated with BCG: a retrospective cohort study. Cell Rep. Med. 1 (5), 100073. doi: 10.1016/j.xcrm.2020.100073 32838341 PMC7405881

[B41] Morton D. L. Eilber F. R. Holmes E. C. Hunt J. S. Ketcham A. S. Silverstein M. J.. (1974). BCG immunotherapy of Malignant melanoma: summary of a seven-year experience. Ann. Surg. 180 (4), 635–643. doi: 10.1097/00000658-197410000-00029 4412271 PMC1344159

[B42] Mouhoub E. Domenech P. Ndao M. Reed M. B. (2021). The diverse applications of recombinant BCG-based vaccines to target infectious diseases other than tuberculosis: an overview. Front. Microbiol. 12. doi: 10.3389/fmicb.2021.757858 PMC8566895 34745066

[B43] Nascimento I. P. Dias W. O. Mazzantini R. P. Miyaji E. N. Gamberini M. Quintilio W.. (2000). Recombinant Mycobacterium bovis BCG expressing pertussis toxin subunit S1 induces protection against an intracerebral challenge with live Bordetella pertussis in mice. Infect. Immun. 68 (9), 4877–4883. doi: 10.1128/iai.68.9.4877-4883.2000 10948100 PMC101688

[B44] Old L. J. Clarke D. A. Benacerraf B. (1959). Effect of Bacillus Calmette-Guerin infection on transplanted tumours in the mouse. Nature 184 (Suppl 5), 291–292. doi: 10.1038/184291a0 14428599

[B45] Palavecino C. E. Céspedes P. F. Gómez R. S. Kalergis A. M. Bueno S. M. (2014). Immunization with a recombinant bacillus Calmette-Guerin strain confers protective Th1 immunity against the human metapneumovirus. J. Immunol. 192 (1), 214–223. doi: 10.4049/jimmunol.1300118 24319265

[B46] Philips J. A. Ernst J. D. (2012). Tuberculosis pathogenesis and immunity. Annu. Rev. Pathol. 7, 353–384. doi: 10.1146/annurev-pathol-011811-132458 22054143

[B47] Rey-Jurado E. Soto J. Gálvez N. Kalergis A. M. (2017). A safe and efficient BCG vectored vaccine to prevent the disease caused by the human Respiratory Syncytial Virus. Hum. Vaccin Immunother. 13 (9), 2092–2097. doi: 10.1080/21645515.2017.1334026 28598702 PMC5612508

[B48] Rivas M. N. Ebinger J. E. Wu M. Sun N. Braun J. Sobhani K.. (2021). BCG vaccination history associates with decreased SARS-CoV-2 seroprevalence across a diverse cohort of health care workers. J. Clin. Invest. 131 (2):e145157. doi: 10.1172/jci145157 33211672 PMC7810479

[B49] Ronning D. R. Vissa V. Besra G. S. Belisle J. T. Sacchettini J. C. (2004). Mycobacterium tuberculosis antigen 85A and 85C structures confirm binding orientation and conserved substrate specificity. J. Biol. Chem. 279 (35), 36771–36777. doi: 10.1074/jbc.M400811200 15192106

[B50] Sadoff J. Gray G. Vandebosch A. Cárdenas V. Shukarev G. Grinsztejn B.. (2021). Safety and efficacy of single-dose ad26.COV2.S vaccine against covid-19. N Engl. J. Med. 384 (23), 2187–2201. doi: 10.1056/NEJMoa2101544 33882225 PMC8220996

[B51] Shahabi V. Reyes-Reyes M. Wallecha A. Rivera S. Paterson Y. Maciag P. (2008). Development of a Listeria monocytogenes based vaccine against prostate cancer. Cancer Immunol. Immunother. 57 (9), 1301–1313. doi: 10.1007/s00262-008-0463-z 18273616 PMC11030952

[B52] Sheerin D. Peton N. Vo W. Allison C. C. Wang X. Johnson W. E.. (2022). Immunopathogenic overlap between COVID-19 and tuberculosis identified from transcriptomic meta-analysis and human macrophage infection. iScience 25 (6), 104464. doi: 10.1016/j.isci.2022.104464 35634577 PMC9130411

[B53] Shelley M. D. Wilt T. J. Court J. Coles B. Kynaston H. Mason M. D. (2004). Intravesical bacillus Calmette-Guérin is superior to mitomycin C in reducing tumour recurrence in high-risk superficial bladder cancer: a meta-analysis of randomized trials. BJU Int. 93 (4), 485–490. doi: 10.1111/j.1464-410x.2003.04655.x 15008714

[B54] Smith V. L. Cheng Y. Bryant B. R. Schorey J. S. (2017). Exosomes function in antigen presentation during an in *vivo* Mycobacterium tuberculosis infection. Sci. Rep. 7 (1), 43578. doi: 10.1038/srep43578 28262829 PMC5338015

[B55] Stover C. K. Bansal G. P. Hanson M. S. Burlein J. E. Palaszynski S. R. Young J. F.. (1993). Protective immunity elicited by recombinant bacille Calmette-Guerin (BCG) expressing outer surface protein A (OspA) lipoprotein: a candidate Lyme disease vaccine. J. Exp. Med. 178 (1), 197–209. doi: 10.1084/jem.178.1.197 8315378 PMC2191093

[B56] Sugawara I. Li Z. Sun L. Udagawa T. Taniyama T. (2007a). Recombinant BCG Tokyo (Ag85A) protects cynomolgus monkeys (Macaca fascicularis) infected with H37Rv Mycobacterium tuberculosis. Tuberculosis (Edinb) 87 (6), 518–525. doi: 10.1016/j.tube.2007.06.002 17720625

[B57] Sugawara I. Sun L. Mizuno S. Taniyama T. (2009). Protective efficacy of recombinant BCG Tokyo (Ag85A) in rhesus monkeys (Macaca mulatta) infected intratracheally with H37Rv Mycobacterium tuberculosis. Tuberculosis (Edinb) 89 (1), 62–67. doi: 10.1016/j.tube.2008.09.008 19028143

[B58] Sugawara I. Udagawa T. Taniyama T. (2007b). Protective efficacy of recombinant (Ag85A) BCG Tokyo with Ag85A peptide boosting against Mycobacterium tuberculosis-infected Guinea pigs in comparison with that of DNA vaccine encoding Ag85A. Tuberculosis (Edinb) 87 (2), 94–101. doi: 10.1016/j.tube.2006.05.001 16815096

[B59] Sylvester R. J. van der M. A. Lamm D. L. (2002). Intravesical bacillus Calmette-Guerin reduces the risk of progression in patients with superficial bladder cancer: a meta-analysis of the published results of randomized clinical trials. J. Urol 168 (5), 1964–1970. doi: 10.1016/s0022-5347(05)64273-5 12394686

[B60] The TB/COVID-19 Global Study Group. (2022). Tuberculosis and COVID-19 co-infection: description of the global cohort. Eur. Respir. J. 59 (3):2102538. doi: 10.1183/13993003.02538-2021 PMC8588566 34764184

[B61] Uno-Furuta S. Matsuo K. Tamaki S. Takamura S. Kamei A. Kuromatsu I.. (2003). Immunization with recombinant Calmette-Guerin bacillus (BCG)-hepatitis C virus (HCV) elicits HCV-specific cytotoxic T lymphocytes in mice. Vaccine 21 (23), 3149–3156. doi: 10.1016/s0264-410x(03)00256-1 12804842

[B62] Usher N. T. Chang S. Howard R. S. Martinez A. Harrison L. H. Santosham M.. (2019). Association of BCG vaccination in childhood with subsequent cancer diagnoses: A 60-year follow-up of a clinical trial. JAMA Netw. Open 2 (9), e1912014. doi: 10.1001/jamanetworkopen.2019.12014 31553471 PMC6763973

[B63] Weng C.-H. Chan P. A. (2020). BCG as an adjunct or alternative vaccine to prevent COVID-19? J. Travel Med. 27 (7):taaa175. doi: 10.1093/jtm/taaa175 PMC7543444 33073845

[B64] WHO. (2021). Global tuberculosis report. (World Health Orgnization).

[B65] Wrapp D. Wang N. Corbett K. S. Goldsmith J. A. Hsieh C. L. Abiona O.. (2020). Cryo-EM structure of the 2019-nCoV spike in the prefusion conformation. Science 367 (6483), 1260–1263. doi: 10.1126/science.abb2507 32075877 PMC7164637

[B66] Yang H. Rao Z. (2021). Structural biology of SARS-CoV-2 and implications for therapeutic development. Nat. Rev. Microbiol. 19 (11), 685–700. doi: 10.1038/s41579-021-00630-8 34535791 PMC8447893

[B67] Zbar B. Rapp H. J. (1974). Immunotherapy of Guinea pig cancer with BCG. Cancer 34 (4 Suppl), 1532–1540. doi: 10.1002/1097-0142(197410)34:8+<1532::aid-cncr2820340827>3.0.co;2-h 4371315

[B68] Zbar B. Tanaka T. (1971). Immunotherapy of cancer: regression of tumors after intralesional injection of living Mycobacterium bovis. Science 172 (3980), 271–273. doi: 10.1126/science.172.3980.271 4323415

[B69] Zhu Y. D. Fennelly G. Miller C. Tarara R. Saxe I. Bloom B.. (1997). Recombinant bacille Calmette-Guérin expressing the measles virus nucleoprotein protects infant rhesus macaques from measles virus pneumonia. J. Infect. Dis. 176 (6), 1445–1453. doi: 10.1086/514140 9395353

